# Supplementary figures and images for: A specific innate immune response silences the virulence of Pseudomonas aeruginosa in a latent infection model in the Drosophila melanogaster host
Source: PLoS Pathog. 2024 Jun 4;20(6):e1012252. doi: 10.1371/journal.ppat.1012252 (PMC11178223; doi:10.1371/journal.ppat.1012252)

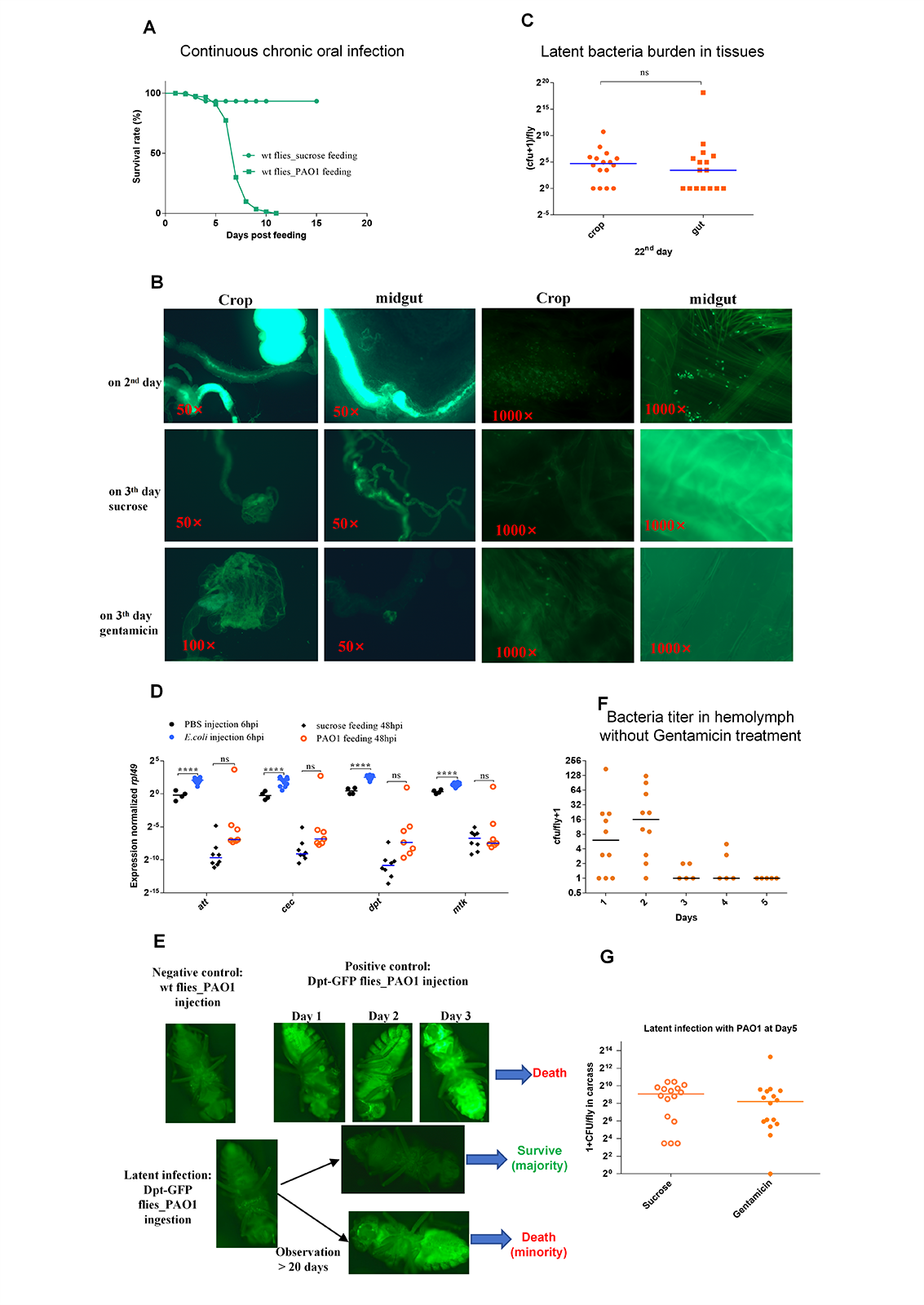

Supplement: S1 Fig — (A) Survival curve of flies feeding continuously on P. aeruginosa PAO1. This experiment was done three times independently, all data were pooled together. (B) clearance of GFP-labeled PAO1 following the ingestion of gentamicin or sucrose solution. The focus is on visceral muscles (right panels). A few bacteria are visible in the crop at high magnification. (C) Bacterial load in digestive tract tissues of latently-infected flies at late stage. Sixteen flies were dissected to pick crops and guts respectively. Bacteria secondarily colonizing the outer part of the digestive tract after having escaped from it likely contribute to the measured bacterial titer. This experiment was only done once. Statistics analysis was done using the t-test. (D) IMD pathway activation measured by RT-qPCR by monitoring the inducibility of imd-regulated AMP genes (att; Attacins; cec: Cecropins; dpt: Diptericin; mtk: Metchnikowin). The level of induction induced by a systemic challenge with Escherichia coli six hours after injection serves as a reference for a full-blown systemic immune response. This experiment was only done once. (E) Visualization by fluorescence microscopy of IMD pathway activation in septic injury systemic infection and latent infection using Diptericin-GFP transgenic reporter flies, the fluorescence was observed under fluorescence microscope. This experiment was performed three times, and a representative one is presented. (F) Bacterial titer in hemolymph of latently infected flies without gentamicin treatment. This experiment was only done once. The bar for each column indicates the median. (G) Bacterial titer in tissue of latently infected flies with or without gentamicin treatment. The bar for each column indicates the median. The t-test was used to assess statistical significance for panels D and G. (TIF) [file ppat.1012252.s001.tif]

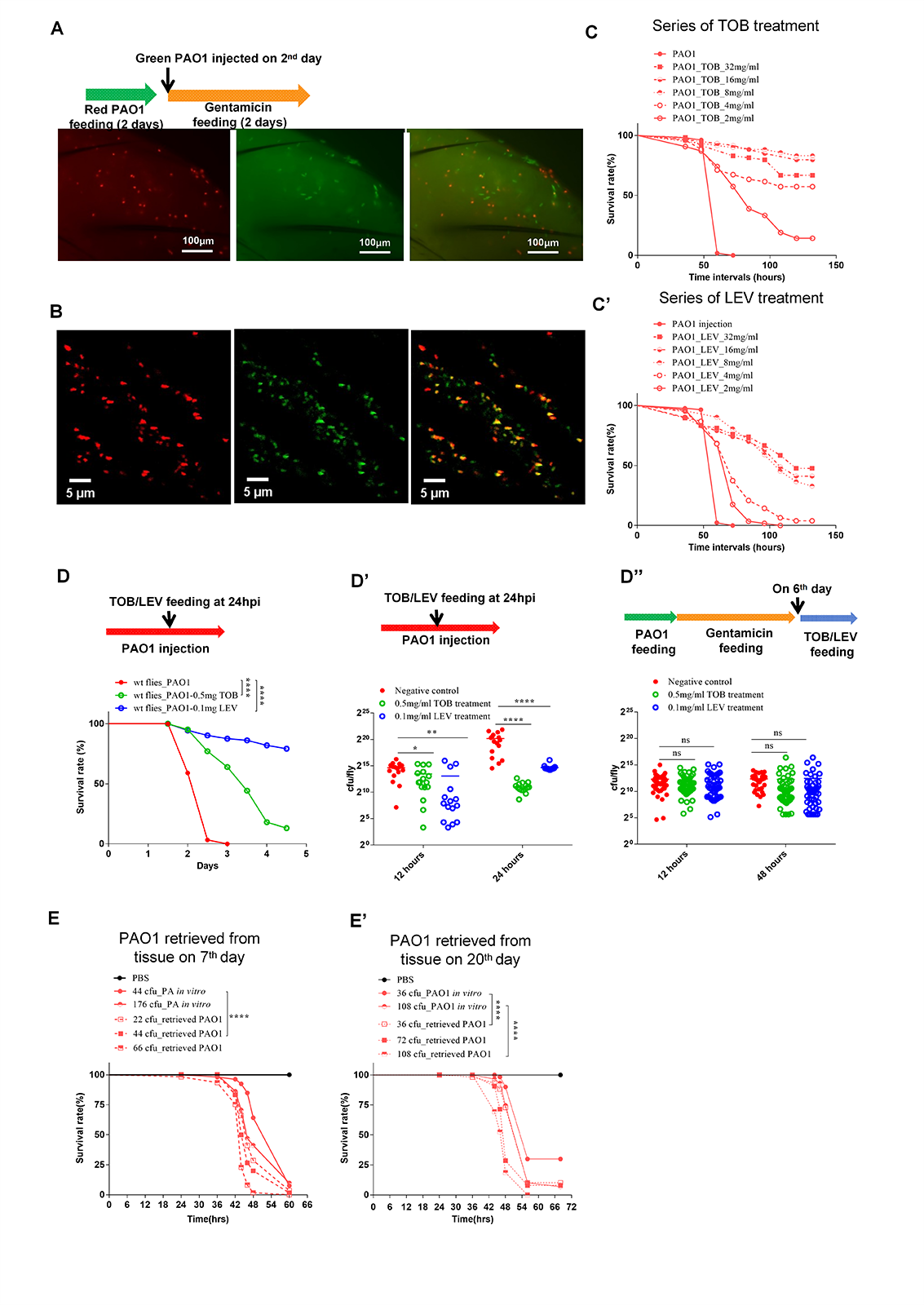

Supplement: S2 Fig — (A) Morphology of P. aeruginosa in distinct infection routes. Flies were fed with OD600 10 RFP-labeled PAO1 for 2 days and then were injected with GFP labeled-PAO1; flies were then dissected two days post injection to observe bacterial morphology under fluorescence microscopy in the same fly. (B) The luminal content of the gut of flies that have ingested PAO1-GFP bacteria was examined. Killed bacteria stained by propidium iodide appear to be red whereas live bacteria are green. (C-C’) P. aeruginosa dose-response inhibition by the injection (4.6 nL) of different doses of Tobramycin (TOB) (C) and of Levofloxacin (LEV) (C’). (D-D”) TOB or LEV per os treatment in P. aeruginosa acute (D and D’) and latent infection (D”). Survival curve of the flies with PAO1 acute injury infection feeding afterwards on TOB and LEV(D). Bacterial titer of PAO1 latently-infected flies feeding on TOB and LEV (D’). Bacterial titer of flies with PAO1 acute injury infection feeding afterwards on TOB and LEV (D”). The bar for each column indicates the median (D’ and D”). (E-E’) Pathogenicity potential of sessile PAO1 in naive flies. P. aeruginosa was isolated from carcass of latently-infected flies at different time points and then injected into naive flies. This experiment was done three times and a representative one is presented here. Statistical analysis was done using Logrank (Mantel-Cox test) in (C-D, E-E’) and Kruskal-Wallis with Dunn’s post-hoc test in (D’-D”). (TIF) [file ppat.1012252.s002.tif]

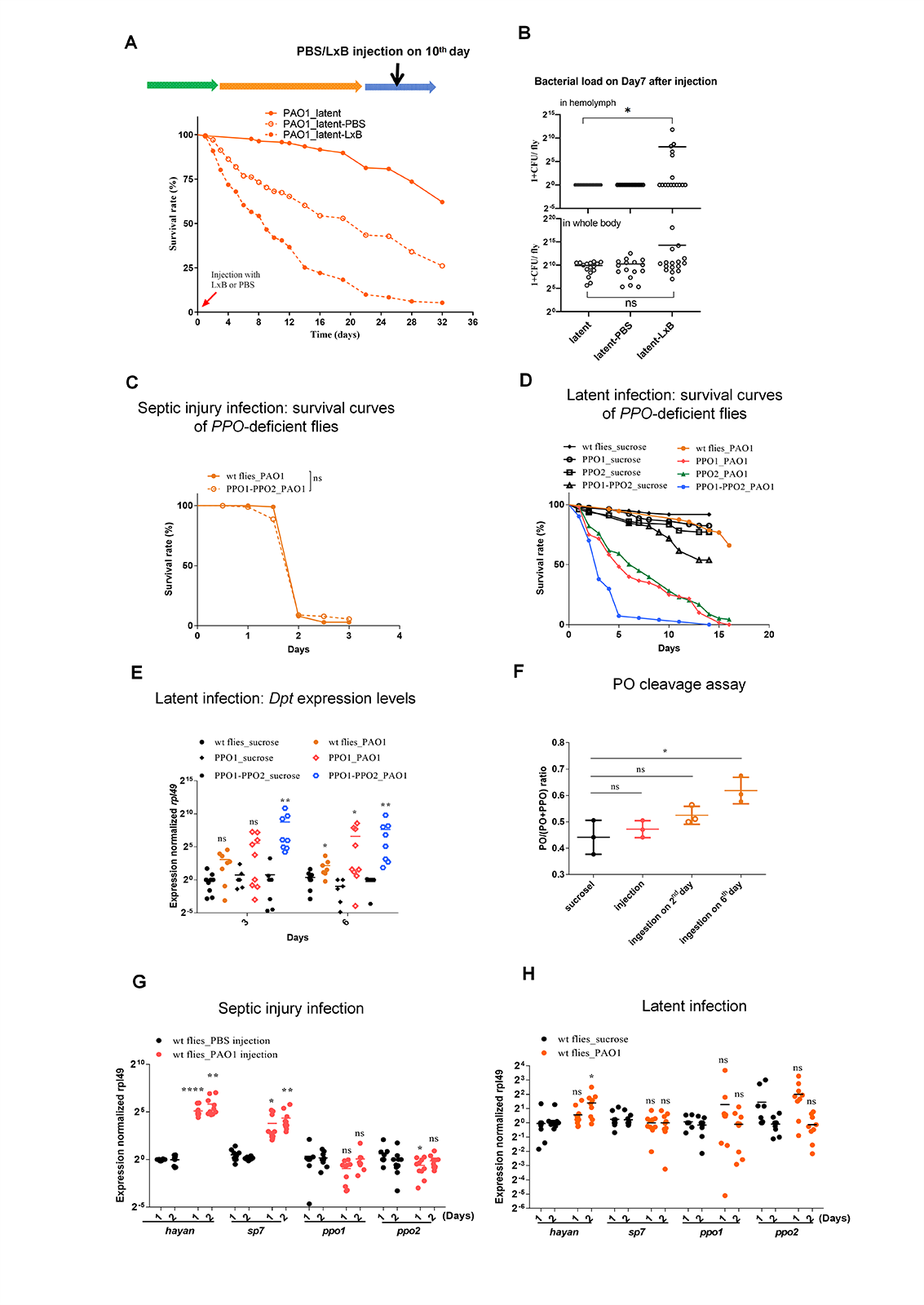

Supplement: S3 Fig — (A) Survival curves of PAO1 latently-infected flies in which the phagocytic abilities of hemocytes have been saturated by the injection of latex-beads that can be phagocytosed but not digested at the indicated time; PBS injection is a control for the effect of injury. LxB indicates latex-beads. Note that the survival curve starts on the day of LxB injection. (B) Bacterial load of PAO1 latently-infected flies with impaired phagocytosis. (C) Survival curves of ΔPPO1-ΔPPO2 immuno-deficient flies after PAO1 acute injury infection. (D) Survival curves of ΔPPO1 or ΔPPO2 immuno-deficient flies after PAO1 latent infection establishing the redundancy of PPO1 and PPO2. (E) Measurement of IMD pathway activation in PPO mutant flies using Diptericin steady-state levels measured by RT-qPCR as a read-out of its activation. (F) Quantification of the intensity of cleavage of PPO into PO in three independent Western blots, one of which is displayed in Fig 3F. (G-H) Expression level of melanization genes measured by RTqPCR after the injection of PAO1 (F) or after PAO1 latent infection (G). The experiments were performed three times and the data were pooled together (A-H). Statistics analysis was done by t-test in (B, E-H), and for each column the bar indicates the mean (B, E, F, H). (TIF) [file ppat.1012252.s003.tif]

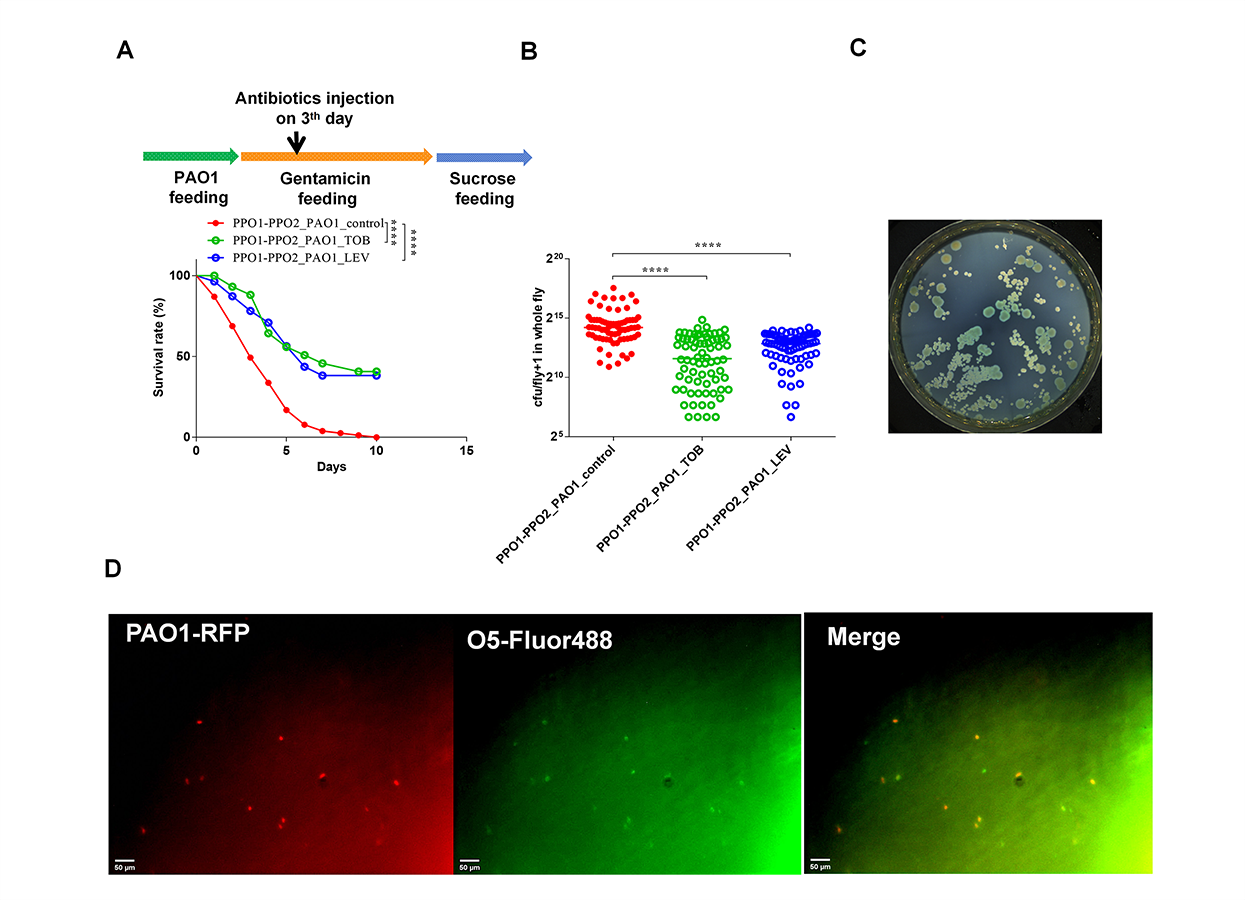

Supplement: S4 Fig — (A-B) Sensitivity of bacteria in vivo to 4.6 nL-injected tobramycin (TOB: 16mg/mL) or levofloxacin (8mg/mL) measured by monitoring the survival (A) and assessing the bacterial load of single whole flies of ΔPPO1-ΔPPO2 immuno-deficient flies (B). Flies were treated with antibiotics on the 3rd day post infection. (C) Colony morphology of PAO1 retrieved from ΔPPO1-ΔPPO2 immuno-deficient flies on 4th day. (D) O5 staining of PAO1 in ΔPPO1-ΔPPO2 immuno-deficient flies. The experiments were performed three times and the data were pooled together (A, B), statistics analysis was done using the Logrank test (Mantel-Cox test) in (A) and the Kruskal-Wallis test with Dunn’s post-hoc test in (B); the bar for each column indicates the median (B). The experiments were performed three times and a representative one is presented here for (C, D). (TIF) [file ppat.1012252.s004.tif]

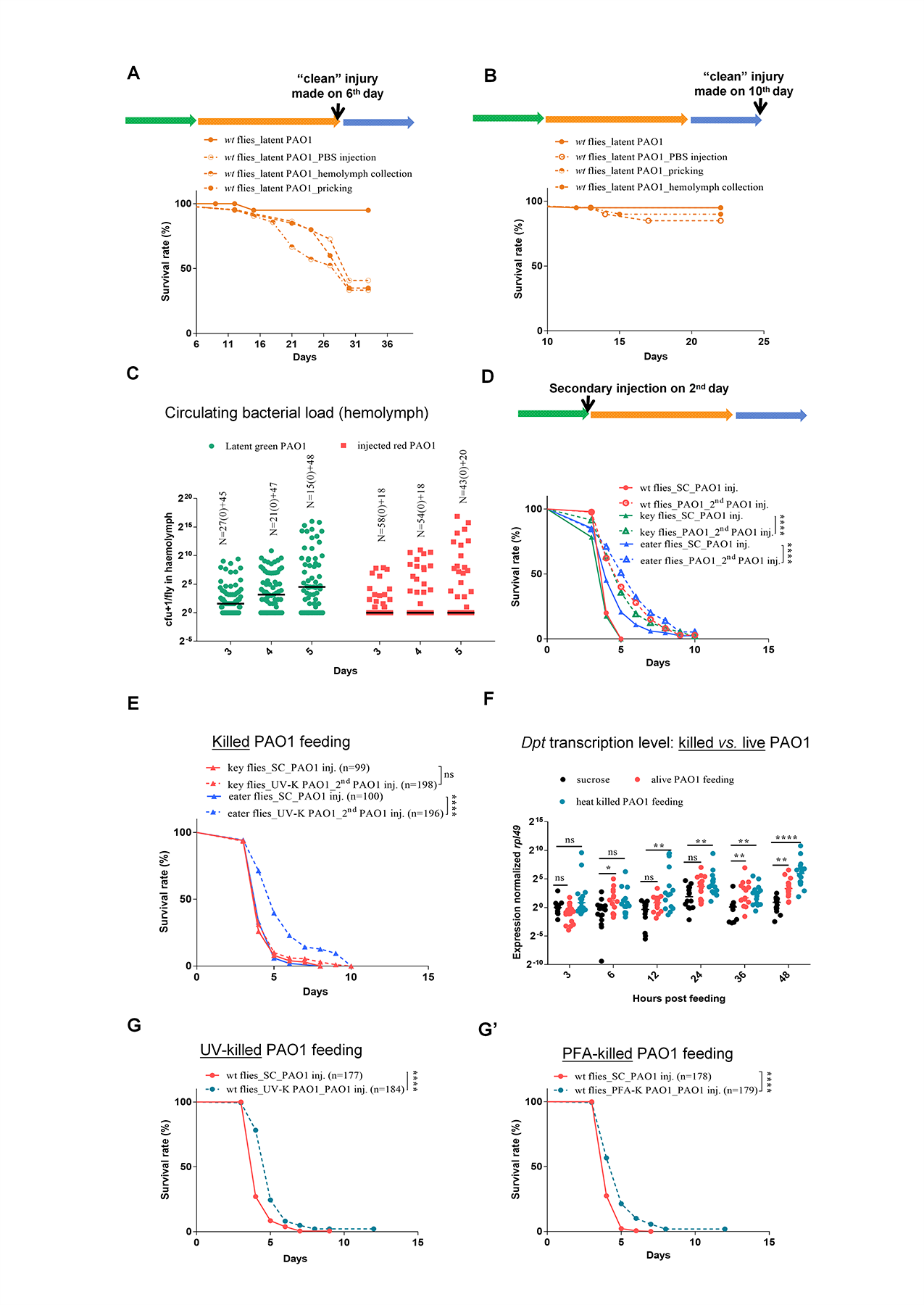

Supplement: S5 Fig — (A-B) Injury of flies at Day 6 or Day 10 activates only partially the virulence of ingested P. aeruginosa. (C) Growth of ingested GFP-labeled PAO1 and injected RFP-labeled PAO1 six days after the beginning of the latent infection protocol. (D) The protection afforded by a latent PAO1 infection against a secondary injection of PAO1 at day 2 of the latent infection protocol is still effective in key- or eater-deficient flies. SC: control flies fed on an uncontaminated sucrose solution (E) The protection afforded by the ingestion of UV-killed bacteria against a secondary PAO1 challenge at day 2 after ingestion requires key but not eater. (F) Whole-flies Diptericin steady-state expression levels as measured by RTqPCR expression level in flies is induced 48 hours after the ingestion of live or killed PAO1 P. aeruginosa. (G-G’) Protection afforded against a secondary PAO1 injection by the ingestion of either UV-killed (G) or PFA-killed PAO1. All the experiments presented here have been performed three times independently and data were pooled together and analyzed by Logrank (Mantel-Cox test) in (A-B, D-E, G-G’), using the Kruskal-Wallis test with Dunn’s post-hoc test in (F); the bar for each column indicates the median (C, F). (TIF) [file ppat.1012252.s005.tif]

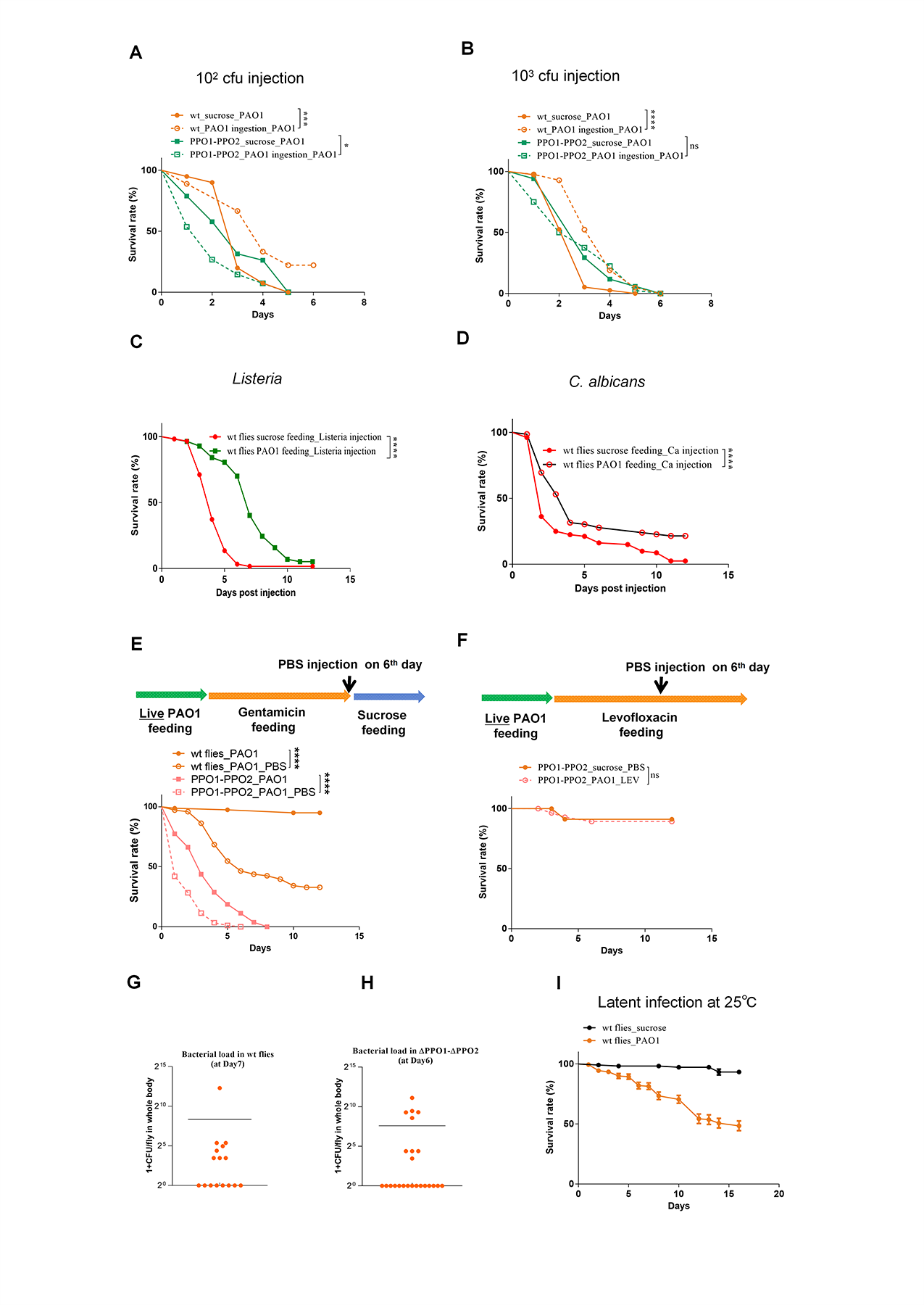

Supplement: S6 Fig — (A-B) Absence of a protective role of ingested P. aeruginosa in ΔPPO1-ΔPPO2 immuno-deficient flies after 102 cfu (A) or 103 cfu (B) PAO1 injection. (C-D) Protective role of ingested P. aeruginosa against Listeria monocytogenes (C) or against Candida albicans systemic infection (D). (E-F) Survival curves of ΔPPO1-ΔPPO2 immuno-deficient flies with PAO1 latent infection by different treatment: PBS injection with gentamicin feeding in (E) and PBS injection with levofloxacin feeding in (F). (G) Bacterial titer of PAO1 ingested for two days by wild-type flies followed by levofloxacin feeding for 5 days. (H) ΔPPO1-ΔPPO2 deficient flies with levofloxacin feeding for 4 days. (I) Survival curve of flies with PAO1 latently-infected performed at 25°C. Flies were fed with 1 OD600 PAO1 suspended in 100mM sucrose solution with 10 percent Brain-Heart Infusion broth for 2 days and then PAO1 in gut lumen were killed by feeding sucrose solution containing 100μg/mL gentamicin for another 4 days, leaving alive only the bacteria that had crossed the digestive tract. Then flies were fed sucrose solution until death. Flies were put at 25°C throughout the infection process. Data have been analyzed using the Logrank test (Mantel-Cox test) in (A-F, I). (TIF) [file ppat.1012252.s006.tif]
