# Supplementary material for: A specific innate immune response silences the virulence of Pseudomonas aeruginosa in a latent infection model in the Drosophila melanogaster host
Source: PLoS Pathog. 2024 Jun 4;20(6):e1012252. doi: 10.1371/journal.ppat.1012252 (PMC11178223; doi:10.1371/journal.ppat.1012252)
Supplement: Attachment — Submitted filename: 141040_1_rebuttal_2925680_s88ttt.pdf [file ppat.1012252.s007.pdf]

Rebuttal for Review Commons to:

"A specific innate immune response silences the virulence of Pseudomonas aeruginosa in a latent infection model in the Drosophila melanogaster host"

We thank the reviewers for their careful scrutiny of our manuscript. We believe that we have addressed satisfactorily the points raised by the reviewers and that our revised manuscript is definitely improved. Our replies below are in blue and use a distinctive font.

Reviewer #1

Evidence, reproducibility and clarity

This works describes a latent Drosophila intestinal infection, which spreads systemically, with a direct systemic Drosophila infection using a common laboratory strain of Pseudomonas aeruginosa. The major observation of this study is that P. aeruginosa can cause a latent infection via its passage through the gut (as opposed to being injected). In doing so it exhibits cell rounding (instead of elongation), reduced cell motility, loss of O5-antigen, antibiotic resistance, ability to cross the intestinal barrier and circulate in the hemolymph and infiltrate the host tissue underneath the cuticle. In addition, latent infection bacteria induce all brunches of the systemic response: the Imd pathway, phagocytosis, and the melanization cascade. Moreover, the melanization pathway protects the host from a secondary systemic infection with various types of bacterial and fungal microbes.

An issue that needs to be clarified is the sensitivity of P. aeruginosa virulence to its biochemical environment. The authors note that. For example, liquid bacterial culture in BHI induces the latent form of bacteria. So the growth conditions and the infection media play a major role in the infection process. They authors need to clarify further the effect of media and infection vehicles, sucrose (high/low), LB, and BHI (as well as temperature) on the latent phenotype.

Temperature is definitely an important parameter and bacteria appear to be somewhat more virulent at 25°C. This point is now addressed in the Material and Methods section (lines 675-679) and in Fig. S6I.

As regards the influence of the composition of the infection solution, it does not seem to be a critical parameter that we have described in the context of continuous feeding on the bacterial solution (Limmer *et al.*, PNAS, 2011). In preliminary experiments, we had tried LB or BHB medium to grow the bacteria and this did not make any difference (see Panel A below for LB [BHB used in our experiments]). As regards the sucrose concentration to the infection solution, we have tried two concentrations and did not observe any difference as regards the establishment of the latent infection. (see panel B below for 50 mM sucrose [100mM used in our experiments]). Of note, *P. aeruginosa* does not grow on sucrose solution alone. However, a latent infection was still established upon feeding the flies with PAO1 in sucrose alone, albeit likely with a mildly increased virulence, in the absence of any BHB medium (see panels C-D) below.

A) *Comparison of LB* vs. *BHB*

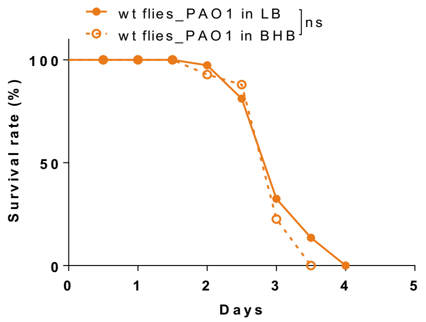

B) *Establishment of latent infection with 50mM sucrose*

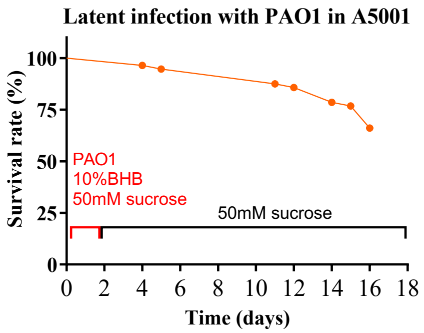

C) *Establishment of a latent infection with a sucrose-only bacterial solution*

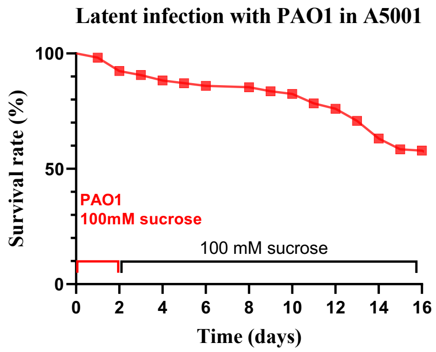

D) *Colonization of host tissues by PAO1 ingested in a sucrose-only bacterial solution*

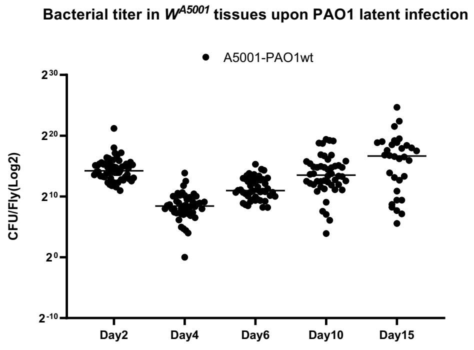

Minor issues:

-Lines 579-581> How were the PAO1GFP/RFP constructed (details are needed)

Done; please, see lines 641-643.

-Figure 1D and other figures > CFUs given as Log2 are unconventional. One cannot easily deduce the burden unless e.g. translate 2e10 to ~1000 and 2e30 to ~10e9 CFUs.

True, but bacterial titer increases by a factor of two at each division cycle. Even though we have previously used a Log10 representation, we now prefer using a Log2 representation. This representation has also been used by other authors in the field, *e.g.,* Duneau *et al*., eLife, 2019.

-Figire S1~~D~~B (now S1C)> "but from the outside of the gut". The given experiment does not prove that statement.

This issue has been already dealt with in the Nehme *et al*. PLoS Pathogens 2007 article, as cited in the manuscript. We further provide in Fig. S1B pictures documenting the presence of bacteria associated with visceral muscles. Finally, we also show that the gut lumen is essentially cleared of bacteria after a period of feeding on a sucrose solution or gentamicin. Hence, most bacterial colonies originate from the outer layer of the gut. We clarify the issue in the text (lines 154-158).

-Lines 146-7 > data are missing in support to the statement.

We have now added Fig. S1B to document that the gentamicin treatment does work, as actually does feeding on sucrose solution alone, as previously documented in Limmer *et al*., 2011 (Fig. S2B). Of note, we cannot exclude that a few bacteria remain, especially in the crop, but those would be at very low titer. Please, see also reply to Reviewer 2.

-Figure S1C > The effect of injury seems to be huge, and may account for much/most of the differences observed (including those between latent and active infection). This is further supported by Figure4A, injury may account for gut collapse and/or systemic stress.

It is well known that injury alone induces the systemic IMD pathway response 6 hours after injury but largely subsides by 24 hours. *The point of Fig. S1C is that the level of induction reached during latent infection is very low as compared to that observed during a systemic infection*, here obtained for reference with an *Escherichia coli* injection and to a lesser extent with a PBS injection. In our latent infection model, we do not perform any injury, except as noted by the reviewer in Fig. 4: the effects of an experimental injury are observed only while the bacteria are crossing the intestinal barrier and hardly any effect is observed when the injury is performed on day 10 (Fig. S4B).

-Figure S1D > How was "fated to die" assessed?

The fluorescent flies were sorted out and their subsequent survival was monitored. As compared to nonfluorescent flies from the same batch, they died within two days of sorting them.

-Figure 3B/10th day > Average line is misplaced.

We thank the reviewer for pointing out this problem. The line is not the average but the median. We have now added a precise description of the bars to all the figure legends.

-Lines 382-5 > what is the evidence of gut damage (or the absence of it)? How do the bacteria escape the gut?

The absence of major gut damages has been documented in Limmer *et al*, PNAS, 2011. How the bacteria escape the gut remains an open question (intracellular and/or paracellular route).

-Lines437-442 > The distinction between dormant P. aeruginosa in the fly tissues and persister cells (upon antibiotic treatment) cannot be justifies on the basis of relative bacterial numbers in the two systems. The extent of resistance to antibiotics though my serve that purpose.

In our latent infection model, most of the bacteria that have crossed the gut barrier become dormant and are associated with tissues, except at the beginning of the infection. In contrast, when a bacterial culture is treated with antibiotics, most of the bacteria are killed by the treatments and only a few ones persist, likely because of an inactive metabolism. Thus, the induction of dormancy in our latent infection model does not rely on the selection of a few metabolically-inactive bacteria able to withstand an immune response or an antibiotic treatment.

Significance

The study is a significant advance to our knowledge.
Notwithstanding further explanations, it provides a solid basis of understanding active versus dormant bacteria.
It further establishes a mode of intestinal to systemic infection as a tool for further explorations.

Reviewer #2

Evidence, reproducibility and clarity

Summary:
In this study, Chen and colleagues investigated a new latent infection model for Pseudomonas aeruginosa using Drosophila melanogaster as a host. First, the authors established a new model for latent Pseudomonas infection. The key feature of this model is the translocation of P. aeruginosa from the gut to the hemolymph and the colonization of fly tissues by the dormant bacteria. Bacteria that translocated from the gut appeared strikingly different in morphology and resistance to antibiotics compared to bacteria that were directly injected. Next, the authors suggest that melanization but not the Imd pathway or hemocytes are necessary to promote dormancy and colonization of fly tissues. Finally, flies with latent P. aeruginosa infection exhibit improved survival after secondary infections in a melanisation-dependent manner. The study reports an interesting model for latent infection, provides insights into the host factors promoting latency and describes some of the consequences of such latent infection for the host. However, some of the conclusions are not fully supported by the data and need further experimental evidence.

Major comments:
1. The latent infection model requires some clarifications. First, temperature. Could the authors explain why they used 18 {degree sign}C and could low temperature contribute to the establishment of dormancy?

As shown in Fig.S6I, the latent infection model is less compelling at 25°C in terms of survival curves, which may reflect an increased rate of spontaneous reactivation of the virulence, a phenomenon we have not studied at 25°C. In another manuscript in preparation (Lin *et al.)*, we actually show that a small heat shock does contribute to reactivation of the bacteria, an issue that is outside of the scope of the present study. Please, see also reply to reviewer 1.

Second, the use of gentamycin. How does gentamicin affect PAO1 outside the gut? From Fig.1C It looks like the cfus in the hemolymph diminished rapidly after gentamicin treatment (around day 3), suggesting the potential effect of the antibiotic. Once the bacteria have crossed the gut and entered the hemolymph, they could still be affected by feeding flies the antibiotic. Is there a possibility that gentamicin treatment is a stress factor that could trigger or facilitate the transition to dormancy? The authors could test this experimentally either by omitting the antibiotic and assessing dormancy or by feeding injected flies with gentamycin and scoring dormancy.

We had actually tested the issue about the potential role of gentamicin outside of the gut compartment. We have thus fed flies on different concentrations of gentamicin and monitored the survival of those flies to the **injection** of PAO1 bacteria (please, see Figure below). When flies were feeding on the highest concentration of gentamicin tested, 32 mg/mL, they were succumbing fast to the PAO1 challenge, but not as fast as nontreated positive control PAO1 injected flies. The use of lower concentrations (16, 8, and 4 mg/mL) led to a progressively stronger protection from PAO1 injection that inversely correlated with the gentamicin dose. We interpret the data with high gentamicin concentrations as an indication that gentamicin at such concentrations is likely directly toxic to the flies, an issue that could be experimentally tested but is not relevant to this study. Interestingly, lower doses led to a much-decreased protection from PAO1 (2mg/mL) to no protection at the dose we use to establish latent infection (100 µg/mL). Thus, these data show that gentamicin can pass the gut barrier when provided at high concentrations, down to 2 mg/mL. However, there is no proof of such a passage at the dose we use. In keeping with this latter possibility, we made a control experiment in which the gentamicin treatment step was replaced by simply feeding on the sucrose solution: the bacterial titer decreased in the hemolymph at the same rate as for gentamicin-treated flies (new Fig. S1F), demonstrating that ingested gentamicin does not contribute to the decreased titer. Rather, the likely depletion of the "source", that is PAO1 in the gut lumen, best accounts for this phenomenon.

We have now cited references which document a lack of permeability of the gut barrier to ingested gentamicin in vertebrate animals (lines 130-133).

As regards the possibility that gentamicin acts as a stress factor on bacteria, our data do not support this possibility, as a latent infection is established in the absence of gentamicin by just feeding the flies on a sucrose solution. We had previously reported that flies fed with *P. aeruginosa* for up to three days do not succumb within the next two weeks when they are fed on a sterile sucrose solution after having ingested the bacterial solution (Limmer *et al.*, PNAS, 2011; Fig. 1C). Under the conditions of two days of PAO1 ingestion, we document in novel Fig. S1G that the carcass is equally well colonized under these conditions.

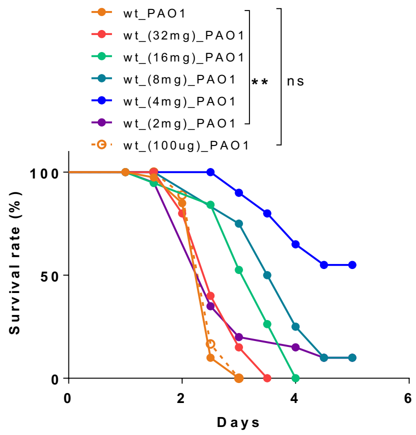

Figure: *impact of gentamicin ingestion at diverse concentrations on the survival of injected PAO1 bacteria.* The ingested antibiotics can act on bacteria present in the hemocoel at concentrations over 2 mg/mL and not at that used in our experiments (100 µg/mL).

2. Does melanization really induce the dormant state of the bacteria? I am not sure the provided data fully support this claim. Addressing these questions might provide a stronger evidence: Fig. 2 A-F: What causes the morphological changes of the bacteria? Melanization or the passage through the gut? Do authors see the same changes in bacteria retrieved from PO-deficient mutant flies? Fig. 2G: Do the authors see the same resistance of PAO1 that colonized PO mutant flies to antibiotics?

In a novel Fig. S4, we now document comprehensively the physiological state of PAO1 bacteria fed to PO-deficient flies. We find that these bacteria are susceptible to antibiotics treatment as they can be rescued from death by the injection of antibiotics on day 3 (Fig. S4A-B). Second, they show a mixed phenotype in terms of colony morphology (Fig. S4C) and bacterial morphology and cell wall properties: even though most bacteria appeared to have kept a rounded morphology, they predominantly (about 75%) expressed the O5-LPS antigen. We interpret these data in terms of a slower transition to virulence than in a septic injury model. Melanization thus strongly contributes to the establishment of latency, even though it is likely that other factors contribute to the establishment of dormancy, but at best provide a minor contribution.

How do PO mutants behave after PAO1 injection? Are they similarly more susceptible?

PPO1-PPO2 mutants are not more susceptible to PAO1 injection than wt controls (new Fig. S3C).

3. Fig. 3F: PPO1 is believed to be the fast-acting PPO, whereas PPO2 is deployed later in infection.

This statement is based on experimental data gained on larvae, not adults. It is not really clear whether the about 10% adult hemocytes that express PPO2 actually contain crystals, in as much as the adult may be better oxygenated than larvae that grow in a hypoxic environment (description by the laboratory of Prof. Jiwon Shim of a role for PO in respiration at the latest EDRC meeting).

How does the Western blot look for PPO1? Will it show an early induction of melanization that could drive the change into the dormant state?

We provide below a characterization of the PPO antibody we use by Western blot analysis. This antibody had originally been raised by the late Dr. Hans-Michael Müller against a PPO from mosquito cell lines, hence explaining its cross-reaction to both *Drosophila* PPO1 and PPO2 (Muller, H.M., Dimopoulos, G., Blass, C., and Kafatos, F.C. (1999)). A hemocyte-like cell line established from the malaria vector Anopheles gambiae expresses six prophenoloxidase genes. J Biol Chem *274*, 11727-11735.). It follows that at least one PO is partially cleaved at day 2 and that both are fully cleaved by day6 of the establishment of the latent infection (Fig. 3F, Fig. S3F).

Figure: *characterization of the antibody raised against* A. gambiae *PPO*

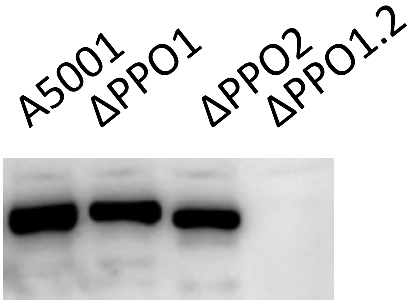

Alternatively, the induction of melanization could also be measured with an L-DOPA test.

This experiment is not needed given the explanation provided above.

4. Fig. 3E: Melanization prevents the growth of PAO1 adhering to tissues, as shown in Fig. 3E. One can see higher levels of cfus in the carcass in PO deficient flies compared to wt flies. However, after, 5 days, there is no difference in the cfus of wt and mutant flies anymore. If the growth inhibition was melanization mediated, would we not expect a consistent growth of bacteria in PO mutants? How to explain the drop in cfus in PO deficient mutants?

This observation is difficult to account for and the explanations we can put forward at this stage are somewhat speculative. It appears that bacteria found in the tissues in PO-deficient flies have a morphology found in *in vitro* culture and within the gut, which does not correlate with virulence but also not with the avirulence state since they are LPS O5 positive. Given the *shallow* survival curves, we envision that there is a progressive release of bacteria from the tissue and then quick proliferation in the hemolymph in a few flies that would then die, but at a frequency too low to reliably ascertain in our hemolymph titer data, with a few flies displaying a high titer (Fig. 3D). By day5, the decreased titer in the carcass may reflect the progressive depletion of tissue-associated bacteria as they progressively become planktonic.

5. Fig. 5D: How do PAO1 bacteria react to Levofloxacin treatment? Do they still go into the dormant state? Do they still attach to tissues? The authors should show that Levofloxacin treatment leads to the same dormant state as gentamycin before interpreting the results of this experiment.

Taken together, our data yield a mixed result. When levofloxacin was fed for two days to latently-infected flies, we found that colonization was not altered (Fig. S2D'), in contrast to a septic injury model in which injected bacteria were susceptible to the ingested antibiotics (Fig. S2D"). Following the reviewer's query, we have further monitored survival and bacterial colonization in the levofloxacin ingestion model. Fig. S2D had already demonstrated that ingested levofloxacin protects the flies from injected PAO1. Fig. S6F shows that the double mutant PO bacteria are protected from ingested PAO1 by the ingestion of this antibiotics. When we monitored the bacterial burden, we found for both wild-type and double PO mutant flies that the bacteria had been cleared in some 50% of the flies. The exact interpretation of the wild-type data is not straightforward. On the one hand, the colonizing bacteria may have become susceptible to the antibiotics even though they remained dormant. On the other hand, they might have been reactivated in their virulence and thus become secondarily susceptible to the antibiotics. For the double PO mutants, the 40% bacteria remaining may witness the mixed bacterial state of PAO1 in these mutants, as documented in Fig. S4. **Nevertheless, the important point is that bacteria are unlikely to contribute to the demise of secondarily infected flies since they have been cleared in at least 50% of the flies, yet the secondarily challenged flies become susceptible only when the relevant melanization genes are affected. The nonPAO1-infected controls succumb faster to the infection than infected ones: the protection against secondary infections is provided by the activation of the melanization cascade by colonizing PAO1 bacteria, even if the colonization is transient in the levofloxacin treatment.**

We have altered the main text to reflect these novel data: lines 387-403.

Minor comments:

Lines 68-72. Mechanisms that are listed are not specific against Gram-negative bacteria but rather general. Please correct.

We are of course aware of this. If it is general, it also applies to Gram-negative bacteria that are the focus of this article. Actually, an earlier version of the manuscript just mentioned the IMD pathway, hence the reference to Gram-negative bacteria. However, the Toll pathway is also required in the host defense against some Gram-negative pathogens such as *P. aeruginosa*. We have now deleted "Gram-negative" in this corrected version.

Line 95. In - as?

We are not sure we understand this comment. We have now added a reference documenting that *P. aeruginosa* can be found in rotting fruits (line 97).

Lines 182-187. Some background information is needed. What is O5 LPS antigen? What motivated the authors to look at it specifically?

The O-antigen is a long-chain polysaccharide motif that constitutes the outermost part of the cell wall. It varies according to the strain. We have added a couple of references that refer to O-antigen (line 198). We had actually already found out this result (unpublished) with the *Serratia marcescens* Db11 O-antigen (O18) that was not found in bacteria that had crossed the gut. The loss of the O5 antigen changes the surface of the bacterium and likely its interactions with tissues and/or the immune system. In the case of *Serratia*, we suspect that the loss of its O-antigen allows the bacterium to be phagocytosed in an *eater*-dependent manner.

Fig. 3C: Why PPO1 and Hayan and PPO1,2 and Sp7 are compared but not mutant vs wild type?

The reason is that it was obviously significant. We have now added the comparisons to wild-type in the revised figure.

How precise is estimation of bacteria in the carcass?

Even though it is not possible to measure how precise these measures are, they are nevertheless reproducible making us confident that they provide an estimate of the rough number of these bacteria found associated to tissues.

How do the authors prevent dissemination of the bacteria during dissection? I wonder if some bacteria might by lost during the dissection (when removing the gut and ovaries) or if you carry over some bacteria from the hemolymph into the carcass measurement? How to make sure, that the bacteria you recover were really adherent and were not leftover from the hemolymph?

It is not possible to prevent dissemination as we cannot fix the tissues and bacteria if we make cfu counts. However, the finding that bacteria are found in the hemolymph only for the first three days, *with a distinct morphology from tissue-associated bacteria,* and not at later time points make us confident that this is not an issue, which suggests that the bacteria are rather tightly attached to the tissues. As regards contamination of tissues by hemolymph, it is also not an issue since the hemolymph titers are so low. However, when the bacteria are actively proliferating to high levels, this is a legitimate concern.

I am also curious how the differences in the cfu levels between whole fly and carcass can be explained (Fig. 1D). After day 5 there are almost no bacteria left in the hemolymph, however, if you compare cfus in the whole fly vs. the carcass, one can see that the whole fly cfus are rising from day 4 onwards. Where do these bacteria come from if not from the hemolymph?

To assess the statement of the reviewer, we now have included the numerical values of the medians of the bacterial burdens displayed in Fig. 1D. There is no increased bacterial burden in whole flies between days 5 to 12; however, the titer is increased at days 15 and 22. Whether this slight increase is biologically relevant is questionable given the spread of the data (see also reply to previous point on the precision of measures). We cannot rigorously exclude that there might be a low-level proliferation of colonizing bacteria late in the latent infection, which has been observed in specific conditions of reactivation of dormant bacteria (Lin *et al., in preparation)*.

Fig. S4D: If the protection to secondary PAO1 infection is not mediated via Imd or phagocytosis, is it

mediated via melanization? How do melanization mutants (increased or decreased) respond to PAO1 secondary infection?

We have performed the experiment (Fig. S6A-B) and found that the protection was abrogated. As noted in the main text, the interpretation is however difficult since the bacteria are no longer in a dormancy state in the PPO mutants.

Significance

This study suggests that host factors, particularly specific immune responses, could drive the latent infections. Hence, besides bacterial mechanisms that received significant attention, we should not underestimate the host's contribution to promoting the latent state in bacteria.
